# PKC-dependent Phosphorylation of the H1 Histamine Receptor Modulates TRPC6 Activity

**DOI:** 10.3390/cells3020247

**Published:** 2014-04-04

**Authors:** Xingjuan Chen, Christian Egly, Ashley M. Riley, Wennan Li, Paul Tewson, Thomas E. Hughes, Anne Marie Quinn, Alexander G. Obukhov

**Affiliations:** 1 Indiana University School of Medicine, 635 Barnhill Dr., MS360A, Indianapolis, IN 46202, USA; 2 Montana Molecular, Bozeman, MT 59715, USA; 3 Stark Neurosciences Research Institute, Indiana University School of Medicine, Indianapolis, IN 46202, USA

**Keywords:** TRPC6, desensitization, DAG, PKC, H1 receptor

## Abstract

Transient receptor potential canonical 6 (TRPC6) is a cation selective, DAG-regulated, Ca^2+^-permeable channel activated by the agonists of G_q_-protein-coupled heptahelical receptors. Dysfunctions of TRPC6 are implicated in the pathogenesis of various cardiovascular and kidney conditions such as vasospasm and glomerulosclerosis. When stimulated by agonists of the histamine H1 receptor (H1R), TRPC6 activity decays to the baseline despite the continuous presence of the agonist. In this study, we examined whether H1R desensitization contributes to regulating the decay rate of TRPC6 activity upon receptor stimulation. We employed the HEK expression system and a biosensor allowing us to simultaneously detect the changes in intracellular diacylglycerol (DAG) and Ca^2+^ concentrations. We found that the histamine-induced DAG response was biphasic, in which a transient peak was followed by maintained elevated plateau, suggesting that desensitization of H1R takes place in the presence of histamine. The application of PKC inhibitor Gö6983 slowed the decay rate of intracellular DAG concentration. Activation of the mouse H1R mutant lacking a putative PKC phosphorylation site, Ser399, responsible for the receptor desensitization, resulted in a prolonged intracellular DAG increase and greater Mn^2+^ influx through the TRPC6 channel. Thus, our data support the hypothesis that PKC-dependent H1R phosphorylation leads to a reduced production of intracellular DAG that contributes to TRPC6 activity regulation.

## 1. Introduction

Transient Receptor Potential Canonical (TRPC) proteins form plasma membrane cation channels that are Ca^2+^-permeable. TRPCs are activated by the agonists of G-protein coupled receptors (GPCRs) in a phospholipase C (PLC) dependent manner. There are seven members in the TRPC subfamily (TRPC1-7). The TRPC6 channel is widely expressed in vascular smooth muscle, where it contributes to regulating vascular tone [1], and the kidney, where it is localized to the glomerular podocyte “split diaphragm” complex [1,2]. Increased expression of TRPC6 was implicated in the pathogenesis of idiopathic pulmonary arterial hypertension (IPAH) [3,4]. The gain-of-function mutants of TRPC6 are strongly associated with the progression of focal segmental glomerulosclerosis (FSGS) [5]. Besides TRPC6 roles in the cardiovascular systems and the kidney, the channel upregulation was reported in some cancer cell models, such as liver cancer cells [6].

It has been demonstrated that diacylglycerol (DAG) is important for gating the TRPC6 channel. DAG is produced by PLC that breaks down phosphatidylinositol diphosphate (PIP_2_) [7]. Two signaling molecules, DAG and inositol triphosphate, are formed upon PIP_2_ break down by PLC. IP_3_ binds to its receptor, IP_3_R, on the endoplasmic reticulum (ER) and releases Ca^2+^ from the ER, an intracellular Ca^2+^ store. This results in a transient intracellular [Ca^2+^] increase in the stimulated cells. DAG acting in concert with intracellular Ca^2+^-activates protein kinase C (PKC), which then translocates to the plasma membrane, where the kinase phosphorylates various protein including channels and membrane receptors.

The activation of non-selective Ca^2+^-permeable channels, such as TRPCs, contributes to the second sustained phase of receptor-induced intracellular Ca^2+^ increases. When activated by histamine via the H1 receptor (H1R), TRPC6 exhibits robust activity that decays over time despite the continuous presence of the agonist. Several seminal studies implicated the DAG-dependent activation of PKC followed by TRPC6 phosphorylation at the residues Serine448 [8] and Ser768/714 (Ser768 in TRPC6A isoforms and Ser714 in TRPC6B) [9] as a cause of TRPC6 activity decay. However, the role of histamine receptor phosphorylation and inactivation has not been considered so far. Indeed, DAG can regulate the functional activity of a plethora of G-protein coupled receptors. There have been several studies that indicated that serine residues (Ser396, Ser398) in the intracellular domain of H1R are potential phosphorylation sites of PKC and site-directed mutagenesis studies suggested that Ser398 residue was primarily involved in PKC-mediate receptor desensitization [10,11,12,13]. Since phosphorylation of GPCR by various kinases is suggested to be an important step in initiating receptor desensitization [14], we hypothesized that TRPC6 channel inactivation may involve the desensitization of the H1 receptor.

In the present study, we used the human embryonic kidney (HEK) cell expression model to investigate the relationship between the intracellular DAG concentration and TRPC6 activity. We found that the histamine-induced DAG production, assessed by using a recently developed fluorescent biosensor [15], results in negative feedback on H1R itself via a PKC-dependent pathway, indicating that desensitization of H1R takes place during prolonged application of histamine. Our results indicate that the inactivation of TRPC6 channel activity in the presence of histamine might be attributed in part to H1R desensitization.

## 2. Experimental Section

### 2.1. Cell Culture and Transfection

HEK cells (American Type Culture Collection, Manassas, VA, USA) were cultured in the Eagle’s minimum essential medium supplemented with 10% fetal bovine serum. HEK cells were transfected using the Lipofectamine LTX reagent (Invitrogen, Carlsbad, CA, USA) in accordance with the manufacturer’s recommendations. The Downward DAG+R-GECO sensor plasmid and the red fluorescent PIP2 sensor plasmid were a gift from Montana Molecular (Bozeman, MT, USA). The mouse H1 receptor plasmid was a gift from Dr. Jean-Charles Schwartz (Centre de recherche, Bioprojet-Biotech, Saint Gregoire Cedex, France) and the pig TRPC6 plasmid was cloned by us previously [16]. We used the following cDNAs mixtures during the transfection procedures: (1) 0.5 µg DAG+R-GECO sensor plasmid, 0.5 µg H1R, and 2 µg TRPC6 plasmid; (2) 0.5 µg DAG+R-GECO sensor plasmid, 0.5 µg H1R, and 2 µg pcDNA3; (3) 0.5 µg PIP_2_ sensor plasmid [17] and 0.5 µg H1R. The cells were cultured for 24–50 h before the fluorescence imaging experiments were performed.

### 2.2. Fluorescence Imaging

Transfected HEK cells were imaged either directly 30–50 h post-transfection time or loaded with Fura-2AM (4 µM) in PBS containing Ca^2+^ and Mg^2+^ for an hour, followed by a 30 min additional incubation in PBS containing no fluorescence dye. A Till Photonics single-cell fluorescence imaging system equipped with an Andor DU885 charge-coupled device camera (Andor Technology PLC, South Windsor, CT, USA) was used to monitor intracellular Ca^2+^ changes in single Fura-2-loaded HEK cells and sensor-expressing HEK cells. The cells were perfused continuously with the test solutions at a rate of 1.5 mL/min.

### 2.3. Molecular Biology

The single-point mutation in the mouse H1 histamine receptor was introduced using a QuikChange Lightning site-directed mutagenesis kit from Agilent Technologies (Santa Clara, CA, USA) as described in the manufacturer’s instructions. The following primer set was used to construct the mutant: GAGGCTCCGCTCACATGCCAGACAGTATGTG (forward primer) and CACATACTGTCTGGC-ATGTGAGCGGAGCCTC (reverse primer). The mutation was verified by sequencing.

### 2.4. Materials

Eagle’s minimum essential medium and fetal bovine serum for HEK cell culture were purchased from Life Technology (Carlsbad, CA, USA); Histamine, Gö6983 and other chemicals were brought from Sigma-Aldrich (St. Louis, MO, USA). The stock solutions were made in water or DMSO and were diluted to the indicated concentrations with the standard extracellular solutions.

### 2.5. Statistics

The statistical analysis was performed using the SigmaPlot 12 software package. The *t*-test followed by the Mann Whitney Rank Sum Test was used to determine whether there is a statistically significant difference between the tested groups. The significance level was set to <0.05. All of the data were presented as mean ± SEM.

## 3. Results and Discussion

### 3.1. Histamine-induced TRPC6 Activity Decays in the Present of Histamine

The TRPC6 channel mediates Ca^2+^ influx in various physiological cellular systems. It was demonstrated that DAG production is a key event for activating TRPC6 [7]. In this work, we employed a dual fluorescent downward DAG and Ca^2+^ biosensor (downward DAG+R-GECO sensor, DDRG) [15] to examine whether the kinetics of TRPC6-mediated Ca^2+^ influx correlates with DAG intracellular concentration changes. We also used a PIP_2_ sensor to confirm that DAG changes reflect the changes in PIP_2_ hydrolysis as was described elsewhere [17]. Since TRPC6 also allows Mn^2+^ influx, we assessed both Ca^2+^ intracellular transients and Mn^2+^ influx in HEK cells expressing pig TRPC6 using the Mn^2+^ quench approach [7,18].

**Figure 1:**
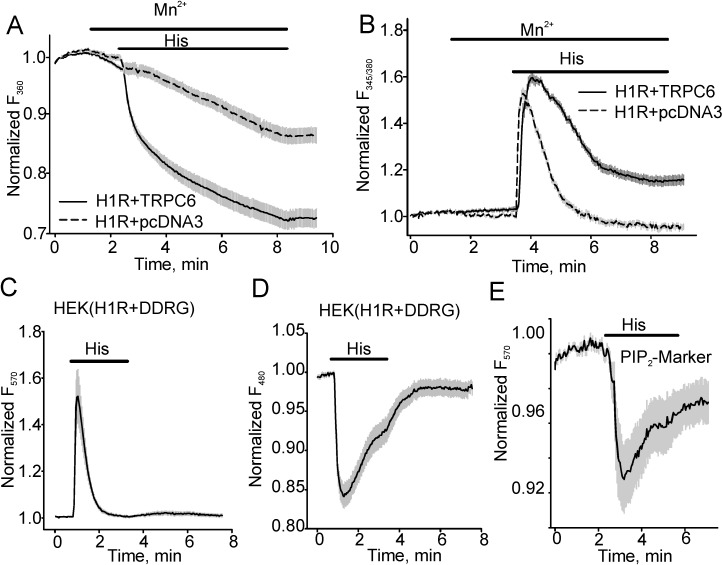
Histamine induces transient intracellular diacylglycerol (DAG), phosphatidylinositol diphosphate (PIP_2_) and Ca^2+^ increases in histamine H1 receptor (H1R)-expressing human embryonic kidney (HEK) cells. (**A**, **B**) The traces show fluorescence changes in HEK cells expressing H1R + pcDNA3 or H1R + TRPC6. Mn^2+^ (0.5 mM) influx was determined in the control group and cells expressing TRPC6 channel using the Mn^2+^ quench approach. Fura-2 fluorescence was excited at 360 nm and the percent of fura-2 fluorescence decrease was shown in (**A**). (**B**) shows fluorescence intensity ratios (F_345_/F_380_) acquired during the same experiment. The fluorescence was normalized to the baseline fluorescence. To detect intracellular Ca^2+^ transients, the downward DAG+R-GECO sensor (DAG/Ca^2+^R) was excited at 570 nm, whereas DAG transients were assessed when DAG+R-GECO sensor fluorescence was excited at 480 nm (**C**, **D**). (**E**) shows the averaged trace (*n* = 12) of PIP_2_ level changes in the presence of histamine in HEK cells co-expressing the red fluorescent PIP_2_ sensor and H1R.

Fura-2 Mn^2+^ quench experiments demonstrated that histamine (40 µM)-induced Mn^2+^ influx was significantly greater in HEK expressing H1R (the percent of Fura-2 quenching was 0.28 ± 0.021, *n* = 26) and TRPC6 than in cells expressing H1R-pcDNA3 (empty plasmid, 0.14 ± 0.017, *n* = 21, Figure 1A). Since Mn^2+^ quench assays the cumulative Mn^2+^ influx through TRPC6, we did not use the approach for kinetics studies. Instead, we investigated the kinetics of intracellular Ca^2+^ transients that were consistently larger in TRPC6 expressing cells as compared to pcDNA3-expressing control HEK cells. As noted by many previous studies [7], we observed that during the continuous presence of histamine, the intracellular [Ca^2+^] increased to a peak at about 30 s after histamine application and subsequently declined slowly with a time constant of 43.89 ± 5.265 s (*n* = 18; Figure 1B). To avoid potential interference due to TRPC6-mediated intracellular Ca^2+^ changes, we next examined intracellular DAG/Ca^2+^ changes in HEK cells expressing H1R and a novel fluorescent dual downward DAG/Ca^2+^ sensor. The downward DAG/Ca^2+^ sensor is a fusion of a PKCδ fragment with circular permuted enhanced green fluorescent protein (GFP) combined by a 2A peptide sequence with a red fluorescent Ca^2+^ sensor R-GECO in one plasmid for stoichiometric co-expression of the sensors ([15], Figure 1C, D). The downward DAG+R-GECO sensor (DAG/Ca^2+^R) was excited at 570 nm for detecting intracellular Ca^2+^ transients and at 480 nm for detecting DAG. Figure 1C, D showed Ca^2+^ changes (Figure 1C, excited at 570 nm) and DAG transients (Figure 1D, excited at 480 nm). Using this approach, we found that histamine-induced [Ca^2+^]_i_ changes were transient in H1R-expressing HEK cells, reflecting the depletion of the intracellular Ca^2+^ stores. We also determined that the production of DAG was not constant over time and exponentially decayed with a close time constant of 48.61 ± 7.07 s (*n* = 23) as intracellular [Ca^2+^] declined (43.89 ± 5.265 s, *n* = 18), suggesting that H1R desensitization may take place. This result was consistent with the data, which we obtained using the red fluorescent PIP_2_ sensor, a fusion of the pleckstrin homology (PH) domain of PLCδ and dimerization-dependent red fluorescent proteins [17] that showed a slowing of intracellular PIP_2_ hydrolysis despite the continuous presence of histamine in the bath (Figure 1E). Importantly, since DAG activates the TRPC6 channel, decreased production of DAG would result in a reduced TRPC6 activity.

### 3.2. The Effect of PKC Inhibition on TRPC6 Inactivation Rate and Decay of DAG

**Figure 2:**
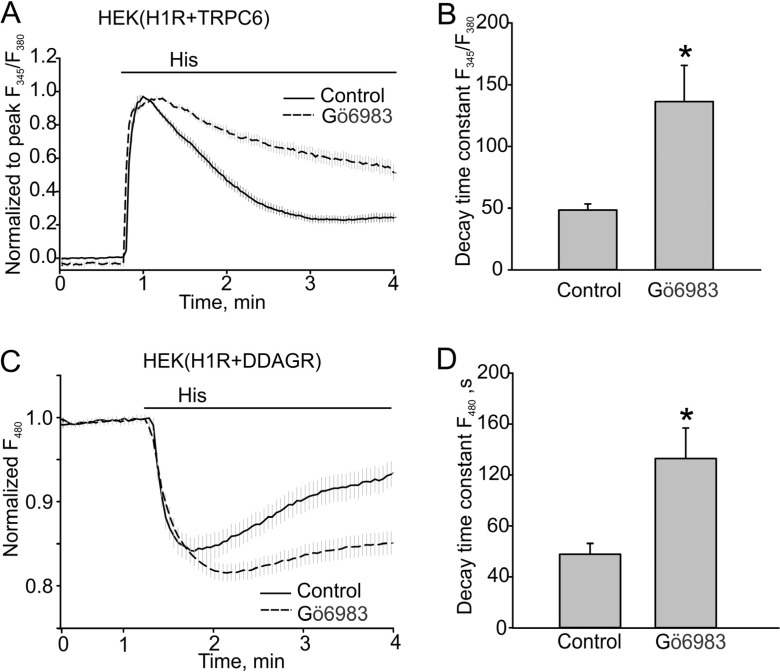
PKC inhibitor Gö6983 slowed the decay of Ca^2+^ and DAG signals in the HEK model. (**A**, **B**) Histamine-induced Ca^2+^ transients in HEK cells expressing H1R + TRPC6 in the absence or presence of PKC inhibitor Gö6983 (500 nM). Fura-2 fluorescence ratios (F_345_/F_380_) are shown. (**A**) Traces of averaged Ca^2+^ transients in the control group and cells treated with Gö6983 (control, *n* = 41 *vs.* Gö6983, *n* = 39). The ratio data were normalized to the peak amplitude following the baseline fluorescence ratio at time zero was subtracted. (**B**) Summary data comparing the decay time constants for the Ca^2+^ transients shown in (**A**). (**C**, **D**) DAG transients assessed with the downwards DAG+R-GECO sensor excited at 480 nm. The fluorescence was normalized to the baseline fluorescence. The bold and broken lines represent the averaged traces, and the vertical gray lines are the SEM values. Data in (**B**, **D**) are presented as mean ± S.E. Asterisk = *p* < 0.05.

It was demonstrated that PKC phosphorylation inactivates TRPC6. Therefore, we first used Gö6983 to confirm whether PKC activity is important for regulating the kinetics of TRPC6 responses. Indeed, we observed that TRPC6 responses decayed much slower in the presence of the PKC inhibitor (48.56 ± 14.77 s, *n* = 41 to 136.30 ± 29.36 s, *n* = 39, *p* < 0.05, Figure 2A, B). Since DAG production also decayed with time, as was determined using the dual DAG/Ca^2+^ sensor, we next investigated whether PKC phosphorylation regulates H1R activity that is known to exhibit PKC-dependent inactivation in other species [19]. We observed that 500 nM Gö6983 also slowed the decay of DAG (48.61 ± 7.07 s, *n* = 17 *vs*. 132.84 ± 23.97 s, *n* = 27, *p* < 0.05, Figure 2C, D).

### 3.3. H1 Receptor Mutant (H1R_S399A_) Maintained A Prolonged DAG Production

**Figure 3:**
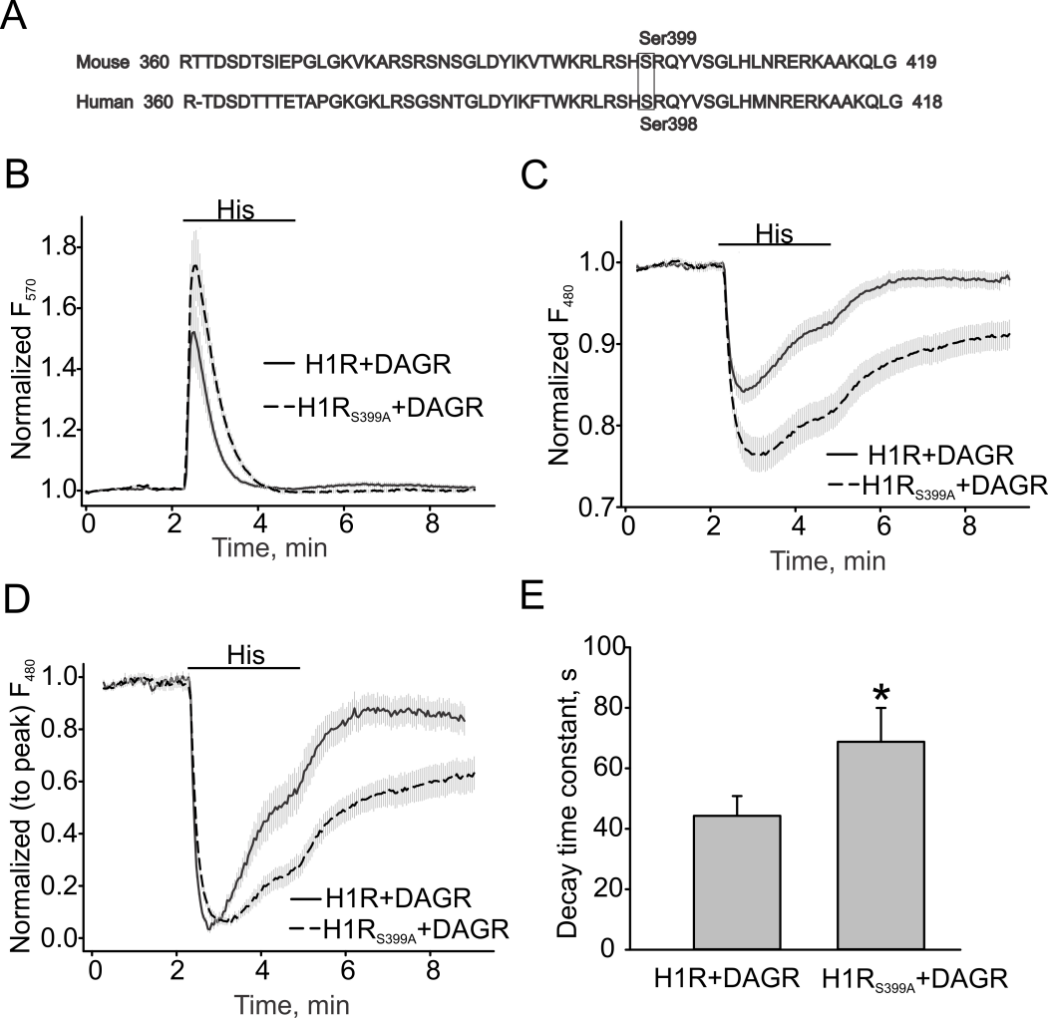
DAG production is more sustained in H1R_S399A_-expressing HEK cells. (**A**) Amino acid sequences and PKC phosphorylation sites in human and mouse H1 receptors. (**B**) Ca^2+^ changes in H1R and H1R_S399A_ expressing HEK cells. The downward DAG+R-GECO sensor (DAG/Ca^2+^R) was excited at 570 nm for detecting intracellular Ca^2+^ transients. (**C**) DAG transients assessed with the downward DAG + R-GECO sensor excited at 480 nm. (**D**, **E**) Summary data on the decay time constants for DAG transients. The bold and broken lines represent the averaged traces, and the vertical gray lines are the SEM values. Averaged traces in H1R and H1R_S399A_ expressing HEK cells are shown as solid and broken lines. Data in (**E**) are presented as mean ± S.E. Asterisk = *p* < 0.05.

In 1998, Fukui’s study provided evidence that ser398 in human H1 receptor was primarily involved in the PKC-mediated desensitization of the receptor [19]. Therefore, we constructed the mouse H1R S399A mutant, H1R_S398A_, lacking a homologous PKC-phosphorylation site (Figure 3A), to investigate whether the mutated receptor would exhibit a slower inactivation rate and more prolonged production of DAG. Figure 3B, C show that the H1R mutant potentiated the Ca^2+^ and DAG responses induced by 40 µM histamine. The normalized DAG response traces and summary data recorded in HEK cells expressing either H1R or its S399A mutant (H1R: 44.31 ± 6.54 s *vs.* S399A: 68.78 ± 11.21 s) are shown in Figure 3D, E.

**Figure 4:**
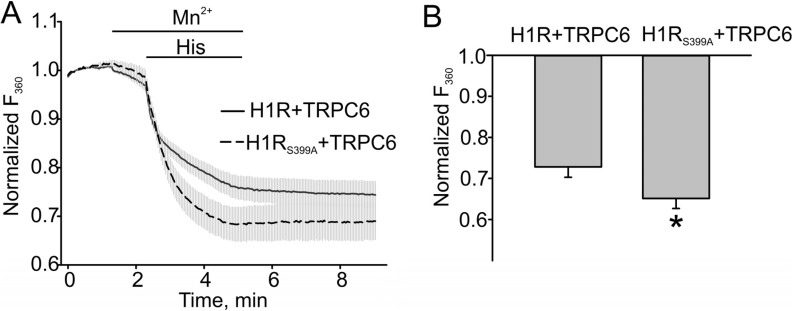
The H1 receptor mutant potentiated the TRPC6 channel activity in HEK model. (**A**) Mn^2+^ (0.5 mM) influx in H1R/TRPC6 expressing HEK cells versus H1R_S399A_/TRPC6 expressing HEK cells, which were measured by the percent of Fura-2 quenching (Fura-2 was excited at 360 nm) at the time point of 5 min. (**B**) The summary data comparing the percent of Fura-2 quenching are shown. The bold and broken lines represent the averaged traces, and the vertical gray lines are the SEM values. Data in (**B**) are presented as mean ± S.E. Asterisk = *p* < 0.05.

We next compared Mn^2+^ influx in HEK cells expressing either H1R and TRPC6 or H1R_S399A_ and TRPC6 to assess the effect of increased DAG production on TRPC6 activity. Indeed, we observed a significantly greater Mn^2+^ influx in the H1R mutant expressing cells, indicating that H1R desensitization results in reduced production of DAG and smaller TRPC6 responses. The summary data are shown in Figure 4B. The percent of fura-2 quenching in H1R and TRPC6 expressing cells was 0.27 ± 0.026, *n* = 17, while that in H1R mutant cells co-expressing the TRPC6 channel was 0.35 ± 0.025, *n* = 18. The influx of Mn^2+^ was significantly increased in cells expressing PKC-phosphorylated deficient H1R mutant (Figure 4, *p* < 0.05).

Thus, we investigated the role of H1R desensitization in regulating TRPC6 activity using the pharmacological and molecular tools. PKC inhibitor Gö6983 slowed the decay of DAG and prolonged TRPC6 activity. The mouse H1 receptor mutant, S399A, which is lacking a critical PKC-phosphorylation site, also potentiated Mn^2+^ influx through TRPC6. However, we also noticed that the effect of Gö6983 was more pronounced on the decay of DAG production and TRPC6 activity as compared to the data obtained with H1R_S399A_. Comparing with Gö6983, the mutant effect only reached a half of the PKC inhibitor efficacy: Gö6983 prolonged the decay time constant from 48.61 ± 7.07 s to 132.84 ± 23.97 s, while the mutant did from 44.31 ± 6.54 s to 68.78 ± 11.21 s. This indicates that PKC phosphorylation of the TRPC6 channel is also a key step for regulating TRPC6 activity. Indeed, it was shown that TRPC6 ser448 is the substrate of PKC [8]. Therefore, Gö6983 not only inhibited receptor phosphorylation but also channel phosphorylation, resulting in a more powerful effect on TRPC6 activity. Our data also suggest that the termination of DAG signaling may be driven, at least in part, by receptor desensitization in addition to the activation of such DAG regulating enzymes as diacylglycerol kinase.

## 4. Conclusions

Here, for the first time, we present evidence that PKC activation negatively regulates TRPC6 activity in part due to H1R desensitization. Our data also support the conclusions of other groups [8,9] that PKC phosphorylation of the TRPC6 protein is important for regulating TRPC6 inactivation rate (see graphic abstract). Increased understanding of the molecular mechanisms underlying TRPC6 channel inactivation may be important for developing novel therapeutic approaches for treating TRPC6 associated dysfunctions. This study also demonstrates that the novel dual DAG/Ca^2+^ and the red fluorescent PIP_2_ sensors are useful tools for studying PLC dependent pathways in native cells.
